# Editorial: New perspectives in robotic-assisted thoracic surgery (RATS)

**DOI:** 10.3389/fsurg.2025.1574220

**Published:** 2025-03-18

**Authors:** Davide Tosi, Edoardo Bottoni, Alessandro Palleschi

**Affiliations:** ^1^ Foundation IRCCS Ca'Granda Ospedale Maggiore Policlinico, Milan, Italy; ^2^ Humanitas Research Hospital, Rozzano, Italy; ^3^ Department of Pathophysiology and Transplantation, University of Milan, Milan, Italy

**Keywords:** video assisted thoracic surgery (VATS), robotic assisted thoracic surgery (RATS), esophagectomy, thymectomy, lung cancer

**Editorial on the Research Topic**
New perspectives in robotic-assisted thoracic surgery (RATS)

In recent decades, robotic-assisted thoracic surgery (RATS) has evolved from a niche innovation to a widely adopted technique across surgical disciplines. Thoracic surgery, in particular, has benefited greatly from the introduction of robotic platforms. Compared to traditional open surgery or video-assisted thoracoscopic surgery (VATS), RATS offers unparalleled precision, superior dexterity, and enhanced visualization through three-dimensional, high-definition optics. However, the adoption of RATS has not been without challenges. Early barriers included high costs, steep learning curves, and limited access to robotic systems. Today, with increased availability, refined training programs, and growing evidence of its clinical benefits, RATS could become the gold standard for a variety of thoracic procedures. This special issue, *New Perspectives in Robotic-Assisted Thoracic Surgery (RATS)*, explores current trends, challenges, and future directions in this innovative field.

## Highlights from the special issue

This special issue features four exemplary articles that illuminate the current state and future potential of RATS and related minimally invasive techniques:

1. *The Impact of Surgical Assistants on Postoperative Complications in Robot-Assisted Ivor-Lewis Esophagectomy for Esophageal Carcinoma* by Wang et al. explores the role of surgical assistants in robot-assisted Ivor-Lewis esophagectomy. Their study found no significant differences in overall complications based on the surgical assistant, though pleural effusion rates varied. This research underscores the importance of refining team dynamics and technique to optimize patient outcomes.
2. *Cost-Consequence Analysis of the Enhanced Recovery After Surgery Protocol in Major Lung Resection with Minimally Invasive Technique (VATS)* by Buja et al. evaluates the economic and clinical benefits of implementing ERAS protocols in VATS procedures. The study demonstrates that ERAS significantly reduces costs and hospital stays without compromising clinical outcomes, highlighting its potential as a dominant strategy in thoracic surgery.
3. *Initial Experience with Robotic Technology for Thoracic Surgery Using the da Vinci Xi System in Tibet, China* by Ping et al. shares insights from the introduction of robotic surgery in a high-altitude region. Despite challenges such as late adoption and logistical constraints, the study demonstrates the safety, reliability, and effectiveness of robotic thoracic surgery in this unique environment, paving the way for broader adoption in resource-limited settings.
4. *Ameliorated Chest Drain Wound Closure in Patients Undergoing Uniportal Thoracoscopic Pulmonary Resection* by Chou et al. describes an improved technique for chest drain wound closure that enhances cosmetic outcomes and patient satisfaction. This innovative approach demonstrates the potential for small but impactful changes to improve minimally invasive thoracic surgery.

One of the key themes explored in this special issue is the expansion of the indications for RATS. Traditionally, robotic platforms were employed for relatively straightforward procedures, but recent advancements have enabled their application in more complex and technically demanding surgeries. Articles in this issue delve into emerging applications, underscoring the versatility of robotic systems and their potential to handle cases previously deemed unsuitable for minimally invasive techniques.

Moreover, innovation in technology is driving the field forward. The integration of artificial intelligence (AI), 5G network and machine learning into robotic platforms is an area of interest, with the potential to revolutionize surgical planning and intraoperative decision-making. Enhanced imaging modalities, such as near-infrared fluorescence imaging and augmented reality, are further enhancing the surgeon's ability to identify anatomical structures and tumor margins with great accuracy. Contributors to this issue provide insights into how these advancements are reshaping the surgical landscape and improving patient outcomes.

As RATS continues to gain traction, the importance of effective training and credentialing cannot be overstated. This special issue highlights the evolving strategies for training thoracic surgeons in robotic techniques, emphasizing the importance of building surgical proficiency and fostering a multidisciplinary approach to patient care.

The economic implications of RATS remain a topic of ongoing debate. While the initial costs of robotic systems and disposable instruments are significant, studies suggest that the long-term benefits—including reduced complications, shorter recovery times, and lower readmission rates—may offset these expenses. This issue examines the cost-effectiveness of RATS across different healthcare systems and considers strategies to improve access to robotic surgery in resource-limited settings.

Equally important are the ethical considerations surrounding the adoption of RATS. Questions about patient consent, surgeon accountability, and equitable access to robotic surgery are explored. As the field progresses, addressing these issues will be critical to ensuring that technological advancements align with the principles of patient-centered care.

Despite its many benefits, RATS is not without limitations. Challenges such as the lack of tactile feedback, dependence on technology, and variability in surgeon expertise continue to pose obstacles. However, these challenges also present opportunities for further innovation and refinement, such as the development of tactile feedback systems, improved ergonomic design and collaboration between engineers and physicians to optimise robotic platforms.

The future of RATS is bright, with ongoing research and development promising to push the boundaries of what is possible in thoracic surgery. As we look ahead, it is clear that the continued integration of robotics into thoracic surgery will not only enhance surgical capabilities but also transform the patient experience.

In conclusion, this special issue of *New Perspectives in Robotic-Assisted Thoracic Surgery (RATS)* underline progresses that has been made in this field. By bringing together different experiences, we aim to provide a comprehensive overview of the current state of RATS, highlight areas for improvement, and inspire future innovation. We hope that the insights presented here will serve as a valuable resource for clinicians, researchers, and policymakers alike, fostering the continued evolution of RATS.

